# Rethinking child safety in community-based health programmes: A critical response to parents’ perceptions of health coordinators

**DOI:** 10.1016/j.puhip.2026.100760

**Published:** 2026-02-27

**Authors:** Ulfah, Kadek Suhardita, Putu Ari Dharmayanti, I Wayan Widana, Rikas Saputra, Erfan Ramadhani, Sri Datuti, Arizona, Rizky Ananda Pohan, Laily Tiarani Soejanto, I Dewa Ayu Eka Purba Dharma Tari, Rr Dwi Umi Badriyah

**Affiliations:** a Universitas Islam Nusantara Bandung, Indonesia; b Universitas PGRI Mahadewa, Denpasar, Indonesia; c Universitas Pendidikan Ganesha, Indonesia; d Universitas Islam Negeri Raden Fatah Palembang, South Sumatra, Indonesia; e Universitas PGRI Palembang, Palembang, Indonesia; f Universitas Mahasaraswati Denpasar, Indonesia; g Institut Agama Islam Negeri Langsa, Langsa, Aceh, Indonesia; h Universitas PGRI Kanjuruhan Malang, Indonesia

**Keywords:** Child safety, Family-based programmes, Parental perceptions, Health coordinators, Community Health, Trust, Cross-sectoral collaboration, Programme sustainability

Dear Editor,

The article [1] *“If children don't feel safe, they won't come back”* presents compelling findings on parents' perceptions of the role of health coordinators in family-based programmes within marginalized communities. The authors successfully highlight a crucial dimension: children's sense of safety as a prerequisite for participation. The relevance of this issue is evident in today's context, where families worldwide face intertwined challenges of health, security, and social vulnerability. We appreciate the contribution of the article in amplifying parents' voices, yet we also note that the framing of safety is positioned rather narrowly in psychosocial and relational terms. This correspondence aims to broaden the perspective by emphasizing child safety as a multidimensional phenomenon that requires cross-sectoral, adaptive interventions to make family-based programmes more inclusive and sustainable [2].

The central concept we emphasize is that child safety is not only individual but also structural, cultural, and systemic. In today's global era, adaptability of family-based health programmes is increasingly urgent, given the diversity of social and cultural contexts [3]. Parents' trust in health coordinators is shaped by local factors such as community norms, service accessibility, and histories of marginalization. The findings in the article are an important foundation, yet generalizability is limited by the specificity of the studied community [4]. Therefore, the discourse on child safety must take into account cross-context flexibility and responsiveness to social dynamics. This would enable family programmes not to become mere policy replications but rather adaptive strategies that strengthen social resilience and public health across diverse settings.

This study makes a valuable contribution by showing how parental perceptions influence programme sustainability. However, a significant gap remains in exploring structural dimensions for example, how economic conditions and public policy shape children's sense of safety [5]. The consequence is that if attention is confined to interpersonal interactions, programmes risk failing to achieve long-term sustainability. On the other hand, the focus on relationships with health coordinators opens positive opportunities: fostering social trust and strengthening community networks. The design of programme platforms, responsive service features, and participatory mechanisms will be decisive for long-term effectiveness. Future research should broaden its scope, exploring policy integration and testing multilevel interventions to ensure that child safety is deeply embedded in community practice [6].

We propose a roadmap consisting of four major steps. First, broaden the definition of child safety to include physical, emotional, social, and structural dimensions. Second, strengthen the capacity of health coordinators by equipping them with cross-cultural competence, contextual sensitivity, and trust-building skills [7]. Third, design participatory monitoring systems involving parents, children, and local communities as active agents. Fourth, reinforce cross-sectoral collaboration through partnerships with government, educational institutions, and civil society organizations. Achieving these goals requires consistent human support from practitioners as well as researchers to build a foundation of trust and sustainable safety [8]. In this way, family-based community programmes can evolve from short-term responses into holistic, preventive, and context-sensitive strategies.

The broader implication of this commentary is the need to redesign family-based health programmes so that they are not only responsive to parental perceptions but also proactive in shaping safe and adaptive environments. The article under discussion is novel in highlighting the dimension of trust, but its strength is confined to a local scope [9]. Its weakness lies in the lack of explicit connections between child safety and systemic or global factors. Moving forward, research should prioritize integrating multi-level approaches, testing cross-cultural interventions, and exploring long-term strategies [10]. The contribution of this correspondence is to add a global perspective, provide a conceptual roadmap, and direct future research toward strengthening child safety as the foundation of family engagement in community health programmes.

## Declaration of no role of funding source

We, the authors of this article, declare that no funding source played a role in developing this research. The entire process and writing of the article was conducted without financial support from any party, thus ensuring our research's independence, objectivity, and integrity.

## CRediT authorship contribution statement

**Ulfah:** Conceptualization, Formal Analysis, Data curation, Supervision, Writing – original draft, Writing – review & editing. **Kadek Suhardita:** Conceptualization, Formal Analysis, Data curation, Supervision, Writing – original draft, Writing – review & editing. **Putu Ari Dharmayanti:** Conceptualization, Formal Analysis, Supervision, Investigation, and Writing – original draft. **I Wayan Widana:** Conceptualization, Formal Analysis, Supervision, Investigation, and Writing – original draft. **Rikas Saputra:** Conceptualization, Formal Analysis, Supervision, Investigation, and Writing – original draft. **Erfan Ramadhani:** Conceptualization, Formal Analysis, Supervision, Investigation, and Writing – original draft. **Sri Datuti:** Conceptualization, Formal Analysis, Supervision, Investigation, and Writing – original draft. **Arizona:** Conceptualization, Formal Analysis, Supervision, Investigation, and Writing – original draft. **Rizky Ananda Pohan:** Conceptualization, Formal Analysis, Supervision, Investigation, and Writing – original draft**. Laily Tiarani Soejanto:** Conceptualization, Formal Analysis, Supervision, Investigation, and Writing – original draft. **I Dewa Ayu Eka Purba Dharma Tari:** Conceptualization, Formal Analysis, Supervision, Investigation, and Writing – original draft. **Rr Dwi Umi Badriyah:** Conceptualization, Formal Analysis, Supervision, Investigation, and Writing – original draft.

## Funding

The authors declare that no funding was received for this paper.

## Declaration of competing interest

The authors declare that they have no known competing financial interests or personal relationships that could have influenced the work reported in this paper.
